# Trauma exposure and depression among frontline health professionals during COVID-19 outbreak in China: the role of intrusive rumination and organizational silence

**DOI:** 10.1186/s12888-022-04011-0

**Published:** 2022-05-31

**Authors:** Chaofan Li, Qiaobing Wu, Debin Gu, Shiguang Ni

**Affiliations:** 1 Centre for Health Management and Policy Research, School of Public Health, Cheeloo College of Medicine, Shandong University, Jinan, 250012 China; 2 NHC Key Lab of Health Economics and Policy Research (Shandong University), Jinan, 250012 China; 3 Department of Applied Social Sciences, The Hong Kong Polytechnic University, Hung Hom, Kowloon, Hong Kong, China; 4 Institute for Hospital Management, Tsinghua University, Shenzhen, 518055 China; 5 Shenzhen International Graduate School, Tsinghua University, Room 403, Information Technology Tower, Tsinghua Campus, Shenzhen University Town, Shenzhen, 518055 Guangdong China

**Keywords:** Trauma exposure, Depression, Intrusive rumination, Organization silence, Healthcare professional, COVID-19

## Abstract

**Background:**

Healthcare professionals bared particularly high risk and stress during the COVID-19 outbreak. Previous studies have demonstrated that healthcare professionals exposed to COVID-19 incurred various affective disorders including depressive symptoms, anxiety, insomnia, and distress. However, the mechanism underlying the relationship between trauma exposure and depressive symptom among frontline hospital staff has yet to be investigated. This study aims to assess the prevalence of depressive symptoms among frontline healthcare professionals in Shenzhen, China, and elucidate the complex relationship among trauma exposure, intrusive rumination, and organizational silence.

**Methods:**

The data of this study were collected through a time-lagged panel questionnaire survey with three rounds of measurements from February 2020 to May 2020 at an infectious disease hospital in Shenzhen, in which all the confirmed cases of COVID-19 patients were accommodated. Based on cluster sampling design, a total of 134 frontline healthcare professionals directly involved in providing diagnosis, treatment, and nursing services for COVID-19 patients completed three times of web survey. The depressive symptom and trauma exposure were measured via the 12-items General Health Questionnaire and the Explosion Exposure Questionnaire respectively. A moderated mediation model examined the complex interplay among the major study variables. Gender and working year were included as control variables.

**Results:**

Trauma exposure was significantly associated with depression in frontline healthcare professionals. Intrusive rumination mediated the effect of trauma exposure on the depressive symptom, which was moderated by organizational silence. Intrusive rumination presented a more substantial impact on depression while organization silence was lower.

**Conclusions:**

This research demonstrates that intrusive rumination and organizational silence are imperative for predicting the depressive symptoms among the frontline healthcare professionals during the COVID-19 pandemic.

**Supplementary Information:**

The online version contains supplementary material available at 10.1186/s12888-022-04011-0.

## Introduction

### Background

The unprecedented COVID-19 pandemic has incurred tremendous threats worldwide to humankind’s physical and mental health. Healthcare professionals (HCP) involved in the COVID-19 pandemic are exposed to an extremely high risk of physical problems and mental issues; who has to work for long hours in high-pressure environments and be exposed to various types of traumas [1, 2]. Studies from China, Italy, and the United States have indicated that healthcare professionals exposed to COVID-19-related working environments have a mental illness, such as depression, anxiety, insomnia, and distress [3–6]. Moreover, compared to healthcare professionals in other positions, frontline hospital staff had a higher likelihood of reporting depressive symptoms [7]. Potential interventions, such as adaptive training, psychological support, assistance from psychologists, and online psychological counseling, could be implemented to help medical staff deal with mental health issues [8]. However, empirical research on the explanatory mechanism linking depression symptoms to trauma exposure among the frontline hospital staff is still relatively scarce. Thus, it calls for more in-depth research to explain when and how frontline hospital staff experienced depressive symptoms, informing targeted and effective psychological interventions or support [9]. To fill the research gap, this study aims to explore the prevalence of depressive symptoms among frontline hospital staff in Shenzhen, the metropolis and border city of China’s mainland and Hong Kong, during the outbreak of COVID-19 and to identify potential mediators and moderators explaining the relationship between trauma exposure and depressive symptoms. Understanding that trauma exposure predicts depression is expected to have important theoretical, practical, and policy implications for developing effective strategies to prevent depression among frontline healthcare professionals.

### Theory and hypotheses

#### Trauma exposure and depression

Depression is a pervasive negative mental health outcome of trauma exposure [10–12]. Many epidemiological studies have reported that depression usually occurs after trauma exposure [13–16]. In addition, depression among frontline healthcare workers dealing with the COVID-19 pandemic was 58.5%, significantly higher than second-line workers (44.8%) [4]. Significantly, The healthcare workers with traumatic events during the COVID-19 pandemic are more likely to suffer from depression, implying that trauma exposure may be a risk factor for depression symptoms. The frontline hospital staff treating COVID-19 patients had to: 1) work for long hours and bear a heavy workload and stress; 2) bear a higher risk of being infected; 3) face the fear of bringing back the virus to household members trying to stay away from family [17]; and 4) accept their incapability to cure confirmed patients [18, 19]. The emergency could constitute a heavy psychological burden and lead to mental illness for frontline healthcare professionals, and hence we hypothesize that:

##### Hypothesis 1

Trauma exposure is positively associated with depression of frontline healthcare professionals during the COVID-19 pandemic.

#### Mediating role of intrusive rumination

Rumination is usually defined as the process or tendency to think repetitively, persistently, and uncontrollably about one’s feelings and problems [20, 21]. It is noteworthy that rumination has several subtypes rather than a unitary construct, including intrusive rumination and deliberate rumination [22, 23]. Intrusive rumination is defined as repetitive and uncontrollable thoughts focused on negative emotions. In contrast, deliberate rumination refers to voluntary and purposeful thoughts that concentrate on understanding events, implications, and problem-solving manners [24].

Rumination is regarded as an indispensable construct to explain and predict the occurrence of depression from both theoretical and empirical aspects [21, 25]. A meta-analysis conducted by Olatunji et al. indicates a robust association between brooding rumination and depression. In contrast, the relations between other subtypes of rumination and depression appeared to be weak [25]. Moreover, several studies investigate the role of rumination in the relationship between trauma exposure and depressive symptom. Nolen illustrates that the ruminative response styles to earthquake trauma exposure could lead to long periods of depression [26]. Ehring reports that intrusive rumination was an essential factor contributing to depression after trauma exposure [27]. Roley also finds that intrusive rumination moderated the relationship between post-traumatic stress disorder and major depression [28].

The response styles theory (RST) proposed by Nolen-Hoeksema explains the relationship between intrusive rumination and depressive symptoms [29]. According to RST, intrusive rumination could exacerbate depression through several cognitive mechanisms: 1) intrusive rumination enhances individuals’ frequency of recalling, understanding, and interpreting his/her trauma exposure circumstances through negative thoughts; 2) intrusive rumination interferes with practical problem-solving actions and instrumental behaviors by thinking in pessimistic and fatalistic ways, which thus aggravates the stressful circumstances; 3) intrusive rumination results in the loss of social support, which in turn fuels the magnitude and prolongs the duration of depressive symptom [21, 26]. Therefore, we hypothesize that:

##### Hypothesis 2

Invasive rumination will medicate the effect of trauma exposure on the depressive symptoms of frontline healthcare professionals during the COVID-19 pandemic.

#### Moderating role of organizational silence in the relationship between trauma exposure and depressive symptom

Organizational silence refers to the behaviors of employees intentionally withholding important opinions, information, or ideas on work-related circumstances or problems [30, 31]. According to the differences in employees’ primary motivation, Vane Dyne categorizes organizational silence into acquiescent, defensive, and prosocial silence [31]. Previous studies identify severe negative consequences of organizational silence in public organizations, such as creating heavy stress and mistrust, lowering the ability to detect errors and misconduct, disengagement, and preventing communications and knowledge sharing [32, 33]. The in-hospital setting, the hierarchical organizational structures, divisions between hospital managers and healthcare professionals, and the lack of cooperation and communications across teams may reinforce hospital organizational silence [34]. Moreover, as Anthony comments, the pressure put on healthcare professionals may raise the issue of organizational silence during the COVID-19 pandemic [35]. Organizational silence at work implicates both employees’ mental health and organizational performance [30]. In terms of personal mental health, psychologists report that inhibiting the expression of emotion and thoughts might influence mental health and psychological functioning [36]. Especially, Morrison proposes that organizational silence could lead to high stress and dissatisfaction [37]. Knoll also finds a negative association between organizational silence and employee mental health and well-being over time [38]. As mentioned above, we develop the third hypothesis as follows:

##### Hypothesis 3

Organizational silence will moderate the associations between trauma exposure and depression through intrusive rumination, and the mediation effects of intrusive rumination will be attenuated when organizational silence is high.

**Fig. 1:**
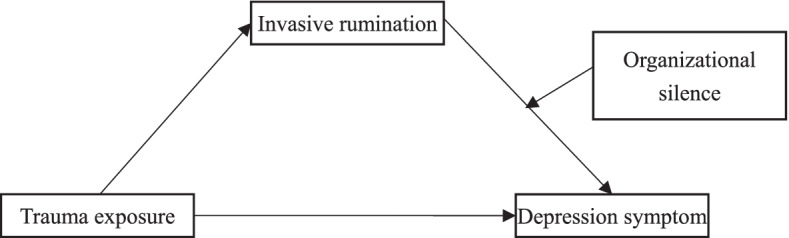
The hypothesized conceptual framework. Notes: It is predicted that trauma exposure has a direct effect on depression symptom and an indirect effect via intrusive rumination. It is assumed that organizational silence moderates the effect of intrusive rumination on depression symptom

The conceptual framework of the study is presented in Fig. 1, in which the mediation role of intrusive rumination in the relationships between trauma exposure and depression and the moderation effect of organizational silence have been illustrated:

## Methods

### Study population

Data for this study were obtained from a time-lagged panel survey with three times of measurements from February 2020 to May 2020. Informed consent was obtained from all the participants. The survey was conducted at an infectious disease hospital in Shenzhen (Hospital T). This hospital accommodated all confirmed COVID-19 cases in Shenzhen during the outbreak and epidemic of COVID-19. The study samples were recruited through a cluster sampling strategy, targeting healthcare professionals directly involved in providing diagnosis, treatment, and nursing care for COVID-19 patients in this hospital. All frontline healthcare professionals coping with COVID-19 were asked to participate in this survey. The baseline (Time 1) data was collected in February 2020, with a total of 492 healthcare professionals completing the survey. Among them, 241 and 117 participants were excluded due to nonresponse at Time 2 and Time 3, respectively. Finally, 134 respondents participated in all three times of surveys, with a 27.24% follow-up response rate. The critical information for all participants is shown in Supplementary Table 1. Our previous study tested for the attrition effects and found no significant differences in gender, age, education level, and marital status among respondents who completed all three surveys and those who dropped out in Time 2 and Time 3 [39].

### Measurement

#### Depressive symptoms

Depressive symptoms were assessed for the 3rd time in the survey via the 12-items General Health Questionnaire (GHQ-12), which is a valuable tool for detecting depression in both the general population and hospital staff. Participants were asked to rate on a 5-point Likert scale ranging from 1 (*never*) to 5 (*always*) on items like how often they felt “constantly under strain,” “unhappy and depressed,” etc. In the study, items 2, 5, 6, 9, 10, and 11 were scored directly, while items 1, 3, 4, 7, 8, and 12 were reverse coded to ensure the direction of each item’s score was consistent. The average value of all the items was calculated to obtain an overall scale score. The Cronbach’s α of the GHQ-12 at Time 3 was 0.88.

#### Trauma exposure

In the baseline survey, we investigated the trauma exposure using the 6-items Explosion Exposure Questionnaire [40] which consisted of two dimensions of trauma exposure: direct exposure and damage. The direct exposure dimension consisted of three items: a) Is any relative confirmed to be a case or died during the COVID-19 pandemic? (*0, no; 1, infected; 2, dead*); b) is there any acquaintance confirmed to be a case or who died during the COVID-19 pandemic? (*0, no; 1, infected; 2, dead*); c) Whether you have witnessed someone infected or dead during the COVID-19 pandemic? (*0, no; 1, yes*). The following three items measured the damage dimension: d) What is the impact of the COVID-19 pandemic on your health status? (*0, none; 1, mild; 2, medium; 3, severe*); e) What is the impact of COVID-19 pandemic on your property? (*0, none; 1, mild; 2, medium; 3, severe*); f) What is the impact of the COVID-19 pandemic on yourself? (*0, none; 1, mild; 2, medium; 3, severe*). The average score of the six items was calculated to form the overall scale score. The Cronbach’s α of the Explosion Exposure Questionnaire in this study was 0.60.

#### Intrusive rumination

In the Time 2 survey, we assessed the intrusive rumination and organizational silence. Three items from the simplified Chinese Version of the Event-Related Rumination Inventory (C-ERRI) were used to measure intrusive rumination [41]. C-ERRI was translated into Chinese by Dong and Liu from its English version and then developed by Cann [24]. As Dong and Cann indicated, C-ERRI had high reliability and validity for measuring intrusive rumination [24, 41]. The original inventory used 10 items to assess intrusive thoughts. In this study, to save responding time of frontline hospital staff, three critical items out of the 10 were selected from C-ERRI to form a short version: a) thoughts about the event distract me or keep me from being able to concentrate; b) other things keep leading me to think about my experience; c) I try not to think about the event, but could not keep the thoughts from my mind. Respondents were asked to rate on a 5-point Likert scale ranging from 1 (*Strongly disagree*) to 5 (*Strongly agree*). The three items’ mean value was considered a score of intrusive rumination. The Cronbach’s α of the short version scale in the study was 0.89.

#### Organizational silence

Organizational silence was assessed by the 6-items Employee Silence Scale (ESS) [42], which comprises three construct dimensions: acquiescent silence, defensive silence, and disregardful silence. Acquiescent silence means that employees keep silent and passively obey orders when they perceive they cannot change the current situation. Two items were selected to measure acquiescent silence: a) The leaders have made decisions, and my opinion would not affect; b) The leaders would not change decisions, and there is no significance even if I express my opinion. Defensive silence refers to employees not expressing their opinions to avoid conflicts and estrangement, which was measured through another two items: c) Remain silence and restraint so as not to be the targets; d) It is unnecessary to offend the leaders and colleagues to express my opinions. Disregardful silence refers to employees not giving any views or opinions because they have a low organizational commitment to the present job or institution. Disregardful silence was assessed by two items: e) I am not concerned about the hospital affairs; f) I choose the “middle way” and will not say anything and bear any more responsibility. Participants were asked to rate six items on a 5-point Likert scale ranging from 1 (*never*) to 5 (*always*). The mean value of the six items was used as a score of organizational silence. The Cronbach’s α of this scale was 0.90.

#### Control variables

Two factors were included as control variables in data analysis, given their potential effects on depressive symptoms. First, gender was controlled as a dichotomous variable (0, male; 1, female). There were gender differences in the prevalence of depression among frontline healthcare professionals [5]. Second, the working experience affected depressive symptoms and organizational silence among doctors and nurses [43, 44]. Therefore, we included years of working in the analysis model and categorized them into four groups: <=5 years, 6—15 years, 16—25 years, and 26 years +.

### Procedure

The survey was conducted online via the Questionnaire Star website during the pandemic of COVID-19. The ethical approval of the survey was obtained from the Institutional Review Board of the Department of Psychology (Ref: 202004), Tsinghua University. All healthcare professionals from that department were invited to complete the online survey. Once they agreed to participate, the questionnaire link was sent to them through WeChat, with informed consent attached to the study. Upon receipt of the completed questionnaires, data could be obtained and downloaded from the server of Questionnaire Star. The baseline survey was conducted in February 2020, followed by the Time 2 and Time 3 surveys in March 2020 and May 2020.

### Data analysis

The hypothesized second stage moderation model was tested using PROCESS v2.15 for SPSS provided by Hayes [45]. All continuous variables were mean-centered before analysis, whereas gender was a binary variable (0, male; 1, female), and working years were categorized into 4 groups (1, <=5 years; 2, 6–15 years; 3, 16–25 years and 4, 26 years +). Three steps were taken to test the hypothesized model: First, an ordinary least squares regression was performed to test the effects of trauma exposure on depressive symptoms; second, a moderated mediation model was tested with intrusive rumination as the mediator and organizational silence as the moderator. To test the significance of the direct and indirect effects, 5000 bootstrap samples were used to calculate the bounds of 95% confidence intervals. Last, we plotted the interactions at three values of organizational silence (*mean* and ± 1 *SD*) to visualize the conditional effects of the moderator.

## Results

### Descriptive statistics and correlations analysis

Table 1 shows the descriptive statistics of all independent and outcome variables and Pearson’s correlation. The negative correlations (− 0.18, − 0.18, and − 0.21, *P* < 0.05) between gender and depression, trauma exposure, and intrusive rumination demonstrated that the male participants reported higher depressive symptoms. Moreover, there were significant positive correlations between working years and intrusive rumination (0.31, *P* < 0.01) and organizational silence (0.17, *P* < 0.05). Trauma exposure, intrusive rumination, and organizational silence were significantly and positively correlated with depressive symptoms. The correlation coefficients were 0.21 (*P* < 0.05), 0.54 (*P* < 0.01) and 0.37 (*P* < 0.01) respectively. Furthermore, trauma exposure, intrusive rumination, and organizational silence were positively associated.

**Table 1:** Descriptive statistics and pairwise correlations among study variables

	*Mean*	*SD*	1	2	3	4	5	6
Gender			1.00					
Male (*N*, %)	40	29.9						
Female (*N*, %)	94	70.1						
Working years	2.09	0.90	−0.10	1.00				
Depression	2.34	0.62	−0.18^*^	0.06	1.00			
Trauma exposure	2.07	0.44	−0.18^*^	0.08	0.21^*^	1.00		
Intrusive rumination	2.42	1.01	−0.21^*^	0.31^**^	0.54^**^	0.33^**^	1.00	
Organizational silence	2.77	0.89	−0.08	0.17^*^	0.37^**^	0.22^*^	0.48^**^	1.00

*N* = 134. ^*^, P < 0.05; ^**^, P < 0.01

### Mediation and moderation analysis

In Step 1, Table 2 shows that trauma exposure was significantly positively associated with depression in healthcare professionals (*β* = 0.18, *P* < 0.05), adjusting for gender and working years. Step 2 showed that trauma exposure was positively correlated with intrusive rumination (*β* = 0.66, *P* < 0.01). Furthermore, intrusive rumination was also positively correlated with depressive symptoms (*β* = 0.31, *P* < 0.01). This supported our second hypothesis that intrusive rumination served as a mediator in the relationship between trauma exposure and depression.

**Table 2:** Results of the moderated mediation

	Step 1			Step 2		
	*β*	*SE*	*t*	*β*	*SE*	*t*
Mediator: Intrusive rumination						
Gender				−0.29	0.17	−1.65
Working year				0.30	0.09	3.41^**^
Trauma exposure				**0.66**	**0.18**	3.64^**^
*R*^*2*^						0.21^**^
Dependent variable: Depression						
Gender	−0.14	0.12	−1.67	−0.12	0.10	−1.19
Working years	0.03	0.06	0.39	−0.07	0.05	−1.30
Trauma exposure	0.18	0.12	2.13^*^	**0.02**	**0.11**	**0.16**
Intrusive rumination				**0.31**	**0.05**	5.67^**^
Organizational silence				**0.10**	**0.06**	**1.78**
Intrusive rumination*Organizational silence				**−0.11**	**0.04**	−2.47^*^
*R*^*2*^			0.26^*^			0.36^**^
∆*R*^*2*^						0.11

*N* = 134. ^*^, P < 0.05; ^**^, P < 0.01

Moreover, organizational silence moderated the association between intrusive rumination and depression. The interaction coefficient between intrusive rumination and organizational silence was − 0.11 (*P* < 0.05), indicating that organizational silence attenuated the positive correlations between intrusive rumination and depression.

**Fig. 2:**
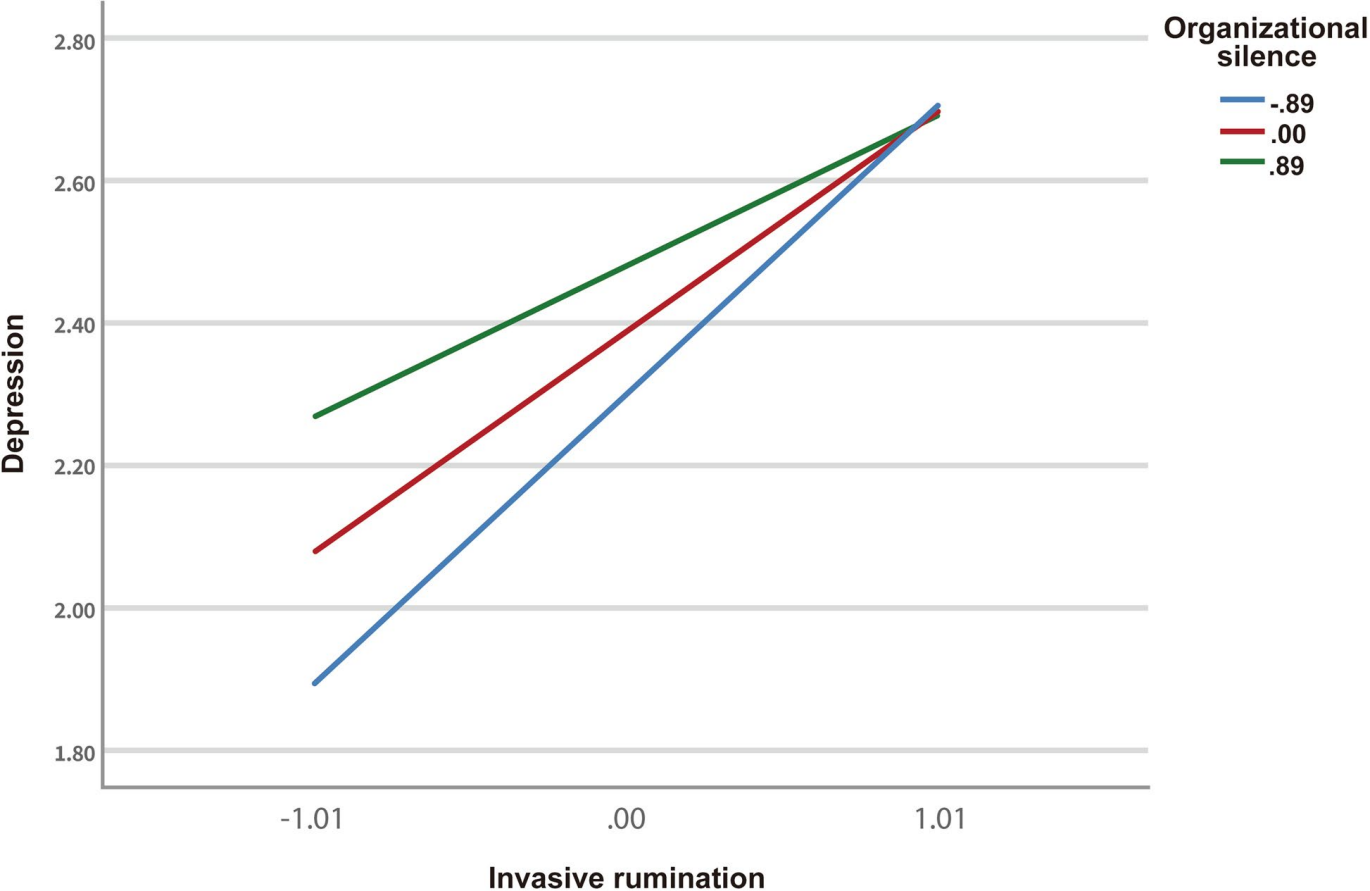
The interaction of Invasive Rumination and Organizational silence. Organizational silence attenuates the indirect effects from trauma exposure to depressive symptom through invasive rumination

Figure 2 shows that intrusive rumination was significantly associated with depression among hospital staff while organizational silence was at either low (*β* = 0.40, *P* < 0.01), medium (*β* = 0.29, *P* < 0.01), or high (*β* = 0.19, *P* < 0.05) level. However, intrusive rumination demonstrated more potent effects on depressive symptoms when organizational silence is lower, indicating a significant moderation effect on the relationship between intrusive rumination and depression in frontline healthcare professionals.

The bootstrap confidence intervals of conditional indirect effect analysis shown in Table 3 were positive. They did not include zero, which implies that the association between trauma exposure and depression was mediated by intrusive rumination regardless of the level of organizational silence. It is noteworthy that the mediation effects of intrusive rumination appeared to be stronger when organizational silence was at a lower level.

**Table 3:** Conditional indirect effect of intrusive rumination on depression at different levels of organizational silence

Mediator	Moderator: Organizational silence	*Effect*	*SE*	*Bootstrap CI*
Invasive rumination	Low	0.27	0.10	[0.11; 0.50]
Invasive rumination	Medium	0.20	0.07	[0.08; 0.38]
Invasive rumination	High	0.14	0.07	[0.04; 0.31]

## Discussion

### Correlations between trauma exposure and depressive symptoms

Consistent with our hypothesis 1, the study results suggested that trauma exposure was positively correlated with depression among hospital staff. This indicated that healthcare professionals at higher risk of exposure to coping with COVID-19 affairs were more likely to suffer from depression. This finding was supported by other cross-sectional studies [4, 19] and a systematic review [5], which concluded that depression and other mental health problems were highly prevalent among the frontline healthcare professionals with exposure to COVID-19 risks or environments. They not only had heave physiological stress to provide diagnosis, treatment, and nursing care but also bear high levels of psychological distress, such as fearing infection or death of family members and friends, worrying about severe damage of COVID-19 to their safety, health, family’s property, and career development. This implies that trauma exposure to long-term physiological and psychological stress are prominent risk factors for depression among healthcare professionals exposed to treating patients with COVID-19.

### The mediation effects of intrusive rumination

In line with our Hypothesis 2, the effect of trauma exposure on depressive symptoms of frontline healthcare professionals was fully medicated by intrusive rumination. This demonstrated that intrusive rumination, as a cognitive mechanism, played an essential role in the process that trauma exposure influenced depressive symptoms. Previous studies have found that intrusive rumination commonly occurs after long-term trauma exposure [27, 46–49]. Furthermore, a previous study argued that intrusive rumination prolonged the period and exacerbated the degree of the adverse effects of trauma exposure [25]. Therefore, it was understandable that individuals exposed to traumatic events with higher levels of intrusive rumination would have more severe depressive symptoms. Consistent with these findings, previous empirical research also proved that intrusive thought could mediate negative effects, such as uncertainty, emotion, and stress, on depressive symptoms [50–54].

### The moderation effect of organizational silence

As for hypothesis 3, this study’s moderated mediation model results suggested that the relationship between intrusive rumination and depressive symptoms was moderated by organizational silence. Similar to previous research findings, this study demonstrated that organizational silence was not only positively associated with depression [55–57] but also with intrusive rumination. Employees who cannot express their ideas and feelings may suffer from high psychological distress, depression, and other mental health problems [37]. In addition, the findings demonstrated that the relationship between intrusive rumination and depression might be varied depending on the levels of organizational silence. The higher level of organizational silence was, the more likely intrusive rumination would significantly affect depressive symptoms. Therefore, if hospital healthcare professionals shared less of their ideas and opinions, intrusive rumination would reinforce their experience of trauma exposure, thereby increasing the prevalence of depressive symptoms.

### Limitations and future research

The results of this research should be interpreted with caution due to several limitations. First, the self-reported measures may result in response and common-method biases [58]. Future research should apply the triangulation approach to collect data from multiple sources and conduct multi-method assessments to measure each psychological concept. Second, participants of this study were concentrated within one hospital, which may influence the representativeness of the sample and the generalizability of the conclusion. Future studies could recruit more representative municipal, provincial or national samples to test the hypothesized model. Third, although there was no significant attrition effect, the non-response rate in the following-up survey was relatively high, which may lead to underestimated bias in trauma exposure and depression prevalence. Fourth, the application of adapted western scales for measuring depression may incur underestimated bias, especially for the Chinese population, who usually reports physical symptoms rather than psychological symptoms [59]. Last, trauma and depression could get assimilated over time [60]. The healthcare professionals exposed to trauma in Time 1 survey may mitigate their distress over time and not report depressive symptoms in Time 3 survey. This time effect may lead to underestimating the total impact of trauma exposure and depression.

### Contributions

Despite these limitations, this study expects to contribute to the literature from both theoretical and practical perspectives. First, the findings shed light on explaining whether and how trauma exposure could predict depressive symptoms. Considering the mediation role of intrusive rumination and moderating effect of organizational silence, this study expands the application of response style theory in interpreting depressive symptoms among a unique population. Second, understanding the explanatory mechanism among trauma exposure, intrusive rumination, organizational silence, and depression also has important implications for designing targeted and appropriate interventions to decrease depressive symptoms among frontline healthcare professionals.

## Conclusion

In summary, this research demonstrates the pivotal role of intrusive rumination and organizational silence in explaining the depressive symptoms among frontline healthcare professionals working in the designated hospital and coping with COVID-19. We expect these research findings to inform psychologists and policy-makers to develop specific interventions or create a more comfortable working environment where hospital staff can benefit from psychological support and alleviate their mental health burden.

## Supplementary Information

**Additional file 1: Supplementary Table 1**. Descriptive statistics of participants.

## Data Availability

The datasets used and/or analyzed during the current study are available from the corresponding author on reasonable request.
