# Supplementary material for: Trauma exposure and depression among frontline health professionals during COVID-19 outbreak in China: the role of intrusive rumination and organizational silence
Source: BMC Psychiatry. 2022 May 31;22:366. doi: 10.1186/s12888-022-04011-0 (PMC9153217; doi:10.1186/s12888-022-04011-0)
Supplement: Supplementary file 1 — Additional file 1: Supplementary Table 1. Descriptive statistics of participants. [file 12888_2022_4011_MOESM1_ESM.docx]

Supplementary Table 1. Descriptive statistics of participants

| Variables | *N* | % |
| --- | --- | --- |
| Age (Mean, Sd) | 35.07 | 8.13 |
| Job type |  |  |
| Doctor | 38 | 28.4 |
| Nurse | 44 | 32.8 |
| Administrator | 33 | 24.6 |
| Other | 19 | 14.2 |
| Education |  |  |
| Junior college | 23 | 17.1 |
| Bachelor | 73 | 54.5 |
| Master and above | 38 | 28.4 |
| Marital status |  |  |
| Unmarried | 33 | 24.6 |
| Married | 95 | 70.9 |
| Divorced, widowed and others | 6 | 4.5 |
| Professional title |  |  |
| No title | 25 | 18.7 |
| Primary | 39 | 29.1 |
| Middle | 47 | 35.1 |
| Senior | 23 | 17.1 |
